# Supplementary material for: A vaccinia-based single vector construct multi-pathogen vaccine protects against both Zika and chikungunya viruses
Source: Nat Commun. 2018 Mar 26;9:1230. doi: 10.1038/s41467-018-03662-6 (PMC5964325; doi:10.1038/s41467-018-03662-6)
Supplement: Supplementary file 1 — Supplementary Information (PDF 2086 kb)(PDF 1150 kb) [file 41467_2018_3662_MOESM1_ESM.pdf]

Supplementary figures

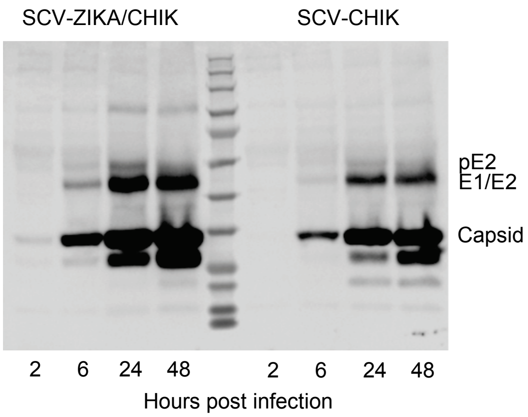

Supplementary Fig. 1. Western blot using mouse polyclonal anti-CHIKV sera (as in Fig. 1e) of lysates of HeLa cells infected with SCV-ZIKA/CHIK or SCV-CHIK (MOI≈5) and harvested at the indicated time points.

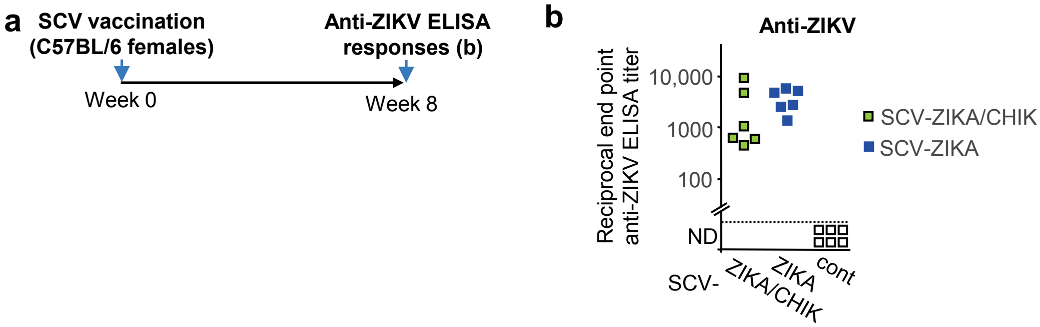

Supplementary Fig. 2. **a** Timeline of vaccination and antibody assays. Mice were vaccinated with SCV-ZIKA/CHIK as in Fig 2a, and the same dose and route of SCV-ZIKA or SCV-cont. **b** IgG ELISA titers determined as in Fig. 2b.

SCV-ZIKA was constructed by replacing the CHIKV structural gene cassette from SCV-ZIKA/CHIK with an EcoGPT/GFP expression cassette, with the EcoGPT/GFP expression cassette subsequently removed by intramolecular recombination.

Supplementary Fig. 3. Nucleotide sequences for the CHIKV and ZIKV immunogen inserts and D13L deletion for SCV-ZIKA/CHIK.

CHIKV immunogen genes located between A38L and A40R ORFs

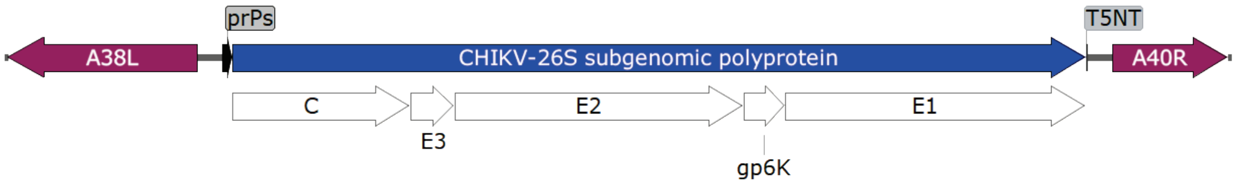


```
LOCUS       Exported                5381 bp ds-DNA     linear   VRL 27-FEB-2018
DEFINITION  CHIKV-26S expression cassette within SCV1002.
ACCESSION   .
VERSION     .
KEYWORDS    .
SOURCE      SCV1002
  ORGANISM  SCV1002
REFERENCE   1  (bases 1 to 5381)
  AUTHORS   Paul Howley
  TITLE     Direct Submission
  JOURNAL   Exported Feb 27, 2018 from SnapGene 4.1.5
            http://www.snapgene.com
COMMENT     CHIKV-26S expression cassette was inserted by homologous
            recombination to replace the A39R ORF
FEATURES             Location/Qualifiers
     source          1..5381
                     /organism="SCV1002"
                     /strain="Copenhagen"
                     /mol_type="genomic DNA"
                     /note="SCV single vectored chikungunga and Zika virus
                     vaccine - CHIKV expression cassette insertion site
                     replacing A39R ORF"
     CDS             complement(13..846)
                     /codon_start=1
                     /product="CD47 antigen/integrin-associated protein"
                     /label=A38L
                     /note="VACV-COP-A38L ORF"
                     /translation="MSRVRISLIYLYTLVVITTTKTIEYTACNDTIIIPCTIDNPTKYI
                     RWKLDNHDILTYNKTSKTTILSKWHTSARLHSLSDSDVSLIIEYKDILPGTYTCEDNTG
                     IKSTVKLVQLHTNWFNDYQTMLMFIFTGITLFLLFLEITYTSISVVFSTNLGILQVFGC
                     VIAMIELCGAFLFYPSMFTLRHIIGLLMMTLPSIFLIITKVFSFWLLCKLSCAVHLIIY
                     YQLAGYILTVLGLGLSLKECVDGTLLLSGLGTIMVSEHFSLLFLVCFPSTQRDYY"
     promoter        957..1000
                     /label=prPs
                     /note="Vaccinia strong early/late promoter"
     CDS             1001..4747
                     /codon_start=1
                     /product="CHIKV 26S subgenomic polyprotein"
                     /label=CHIKV-26S subgenomic polyprotein
                     /note="Chikungunya virus 26S subgenomic polyprotein coding
                     sequence from Reunion strain that adapted to Aedes
                     albopictus mosquito (Genbank: AM258992)"
                     /translation="MEFIPTQTFYNRRYQPRPWTPRPTIQVIRPRPRPQRQAGQLAQLI
                     SAVNKLTMRAVPQQKPRRNRKNKKQKQKQQAPQNNTNQKKQPPKKKPAQKKKKPGRRER
                     MCMKIENDCIFEVKHEGKVTGYACLVGDKVMKPAHVKGTIDNADLAKLAFKRSSKYDLE
                     CAQIPVHMKSDASKFTHEKPEGYYNWHHGAVQYSGGRFTIPTGAGKPGDSGRPIFDNKG
                     RVVAIVLGGANEGARTALSVVTWNKDIVTKITPEGAEEWSLAIPVMCLLANTTFPCSQP
                     PCTPCCYEKEPEETLRMLEDNVMRPGYYQLLQASLTCSPHRQRRSTKDNFNVYKATRPY
                     LAHCPDCGEGHSCHSPVALERIRNEATDGTLKIQVSLQIGIKTDDSHDWTKLRYMDNHM
                     PADAERAGLFVRTSAPCTITGTMGHFILARCPKGETLTVGFTDSRKISHSCTHPFHHDP
```

```
                     PVIGREKFHSRPQHGKELPCSTYVQSTAATTEEIEVHMPPDTPDRTLMSQQSGNVKITV
                     NGQTVRYKCNCGGSNEGLTTTDKVINNCKVDQCHAAVTNHKKWQYNSPLVPRNAELGDR
                     KGKIHIPFPLANVTCRVPKARNPTVTYGKNQVIMLLYPDHPTLLSYRNMGEEPNYQEEW
                     VMHKKEVVLTVPTEGLEVTWGNNEPYKYWPQLSTNGTAHGHPHEIILYYYELYPTMTVV
                     VVSVATFILLSMVGMAAGMCMCARRRCITPYELTPGATVPFLLSLICCIRTAKAATYQE
                     AAIYLWNEQQPLFWLQALIPLAALIVLCNCLRLLPCCCKTLAFLAVMSVGAHTVSAYEH
                     VTVIPNTVGVPYKTLVNRPGYSPMVLEMELLSVTLEPTLSLDYITCEYKTVIPSPYVKC
                     CGTAECKDKNLPDYSCKVFTGVYPFMWGGAYCFCDAENTQLSEAHVEKSESCKTEFASA
                     YRAHTASASAKLRVLYQGNNITVTAYANGDHAVTVKDAKFIVGPMSSAWTPFDNKIVVY
                     KGDVYNMDYPPFGAGRPGQFGDIQSRTPESKDVYANTQLVLQRPAVGTVHVPYSQAPSG
                     FKYWLKERGASLQHTAPFGCQIATNPVRAVNCAVGNMPISIDIPEAAFTRVVDAPSLTD
                     MSCEVPACTHSSDFGGVAIIKYAASKKGKCAVHSMTNAVTIREAEIEVEGNSQLQISFS
                     TALASAEFRVQVCSTQVHCAAECHPPKDHIVNYPASHTTLGVQDISATAMSWVQKITGG
                     VGLVVAVAALILIVVLCVSFSRH"
     mat_peptide     1001..1783
                     /product="Capsid protein"
                     /label=C
                     /note="Chikungunya virus capsid protein"
     mat_peptide     1784..1975
                     /product="glycoprotein E3"
                     /label=E3
                     /note="Chikungunya virus envelope protein"
     mat_peptide     1976..3244
                     /product="glycoprotein E2"
                     /label=E2
                     /note="Chikungunya virus envelope protein"
     mat_peptide     3245..3427
                     /product="glycoprotein 6K protein"
                     /label=gp6K
                     /note="Chikungunya virus envelope protein"
     mat_peptide     3428..4747
                     /product="glycoprotein E1"
                     /label=E1
                     /note="Chikungunya virus envelope protein"
     misc_feature    4748..4754
                     /label=T5NT
                     /note="Pox virus early transcription stop sequence"
     CDS             4866..5372
                     /codon_start=1
                     /product="C-type lectin-like protein; SUMO-1 modified
                     protein; sumoylated protein"
                     /label=A40R
                     /note="VACV-COP-A40R ORF"
                     /translation="MNKPKTDYAGYACCVICGLIVGIIFTATLLKVVERKLVHTPSIDK
                     TIKDAYIREDCPTDWISYNNKCIHLSTDRKTWEEGRNTCKALNPNSDLIKIETPNELSF
                     LRSLRRGYWVGESEILNQTTPYNFIAKNATKNGNIFVAQRILPNCIRVTLYNNYTTFLS
                     YHYFG"
ORIGIN
        1 ttatactcat gcctagtaat agtctctttg cgttgacgga aagcagacta gaaataacag
       61 gctaaaatgt tcagacacca taatagttcc caacccagat aataacagag taccatcaac
      121 acattccttt aaactcaatc ccaaacccaa aaccgttaaa atgtatccgg ccaattgata
      181 gtagataatg aggtgtacag cgcatgataa tttacacagt aaccaaaatg aaaatacttt
      241 agtaattata agaaatatag atggtaacgt catcatcaac aatccaataa tatgccggag
      301 agtaaacatt gacggataaa acaaaaatgc tccgcataac tctatcatgg caataacaca
      361 accaaatact tgtaagattc ctaaattagt agaaaataca acggatatcg atgtataagt
      421 gatctcgaga aataataaga ataaagtaat gcccgtaaag ataaacatca acattgtttg
      481 gtaatcatta aaccaattag tatgaagttg aactaatttc acagtagatt ttattccagt
      541 gttatcctcg catgtataag tacctggtaa gatatcttta tattctataa tcaatgagac
      601 atcactatcc gataacgaat gaagtctagc actagtatgc catttactta atattgtcgt
      661 cttggaagtt ttattataag ttaaaatatc atggttatcc aatttccatc taatatactt
      721 tgtcggatta tctatagtac acggaataat gatggtatca ttacatgctg tatactctat
      781 ggtctttgta gttgttataa caaccaacgt atagaggtat atcaacgata ttctaactct
      841 tgacattttt tatttattta aaatgatacc tttgttattt attttattct attttgctaa
      901 cggtattgaa tggcataagt ttgaaacgag tgaagaaata atttctactt aattaacaaa
      961 aaattgaaat tttatttttt tttttggaa tataaataat atggagttca tcccaaccca
     1021 aactttttac aataggaggt accagcctcg accctggact ccgcgcccta ctatccaagt
     1081 catcaggccc agaccgcgcc ctcagaggca agctgggcaa cttgcccagc tgatctcagc
     1141 agttaataaa ctgacaatgc gcgcggtacc ccaacagaag ccacgcagga atcggaagaa
```

1201 taagaagcaa aagcaaaaac aacaggcgcc acaaaacaac acaaatcaaa agaagcagcc
1261 acctaaaaag aaaccggctc aaaagaaaaa gaagccgggc cgcagagaga ggatgtgcat
1321 gaaaatcgaa aatgattgta ttttcgaagt caagcacgaa ggtaaggtaa caggttacgc
1381 gtgcctggtg ggggacaaag taatgaaacc agcacacgta aaggggacca tcgataacgc
1441 ggacctggcc aaactggcct ttaagcggtc atctaagtat gaccttgaat gcgcgcagat
1501 acccgtgcac atgaagtccg acgcttcgaa gttcacccat gagaaaccgg aggggtacta
1561 caactggcac cacggagcag tacagtactc aggaggccgg ttcaccatcc ctacaggtgc
1621 tggcaaacca ggggacagcg gcagaccgat cttcgacaac aagggacgcg tggtggccat
1681 agtcttagga ggagctaatg aaggagcccg tacagccctc tcggtggtga cctggaataa
1741 agacattgtc actaaaatca cccccgaggg ggccgaagag tggagtcttg ccatcccagt
1801 tatgtgcctg ttggcaaaca ccacgttccc ctgctcccag ccccccttgca cgccctgctg
1861 ctacgaaaag gaaccggagg aaaccctacg catgcttgag gacaacgtca tgagacctgg
1921 gtactatcag ctgctacaag catccttaac atgttctccc caccgccagc gacgcagcac
1981 caaggacaac ttcaatgtct ataaagccac aagaccatac ttagctcact gtcccgactg
2041 tggagaaggg cactcgtgcc atagtcccgt agcactagaa cgcatcagaa atgaagcgac
2101 agacgggacg ctgaaaatcc aggtctcctt gcaaatcgga ataaagacgg atgacagcca
2161 cgattggacc aagctgcgtt atatggacaa ccacatgcca gcagacgcag agagggcggg
2221 gctatttgta agaacatcag caccgtgtac gattactgga acaatgggac acttcatcct
2281 ggcccgatgt ccaaaagggg aaactctgac ggtgggattc actgacagta ggaagattag
2341 tcactcatgt acgcacccat ttcaccacga ccctcctgtg ataggtcggg aaaaattcca
2401 ttcccgaccg cagcacggta aagagctacc ttgcagcacg tacgtgcaga gcaccgccgc
2461 aactaccgag gagatagagg tacacatgcc cccagacacc cctgatcgca cattaatgtc
2521 acaacagtcc ggcaacgtaa agatcacagt caatggccag acggtgcggt acaagtgtaa
2581 ttgcggtggc tcaaatgaag gactaacaac tacagacaaa gtgattaata actgcaaggt
2641 tgatcaatgt catgccgcgg tcaccaatca caaaaagtgg cagtataact cccctctggt
2701 cccgcgtaat gctgaacttg ggaccgaaaa aggaaaaatt cacatcccgt ttccgctggc
2761 aaatgtaaca tgcagggtgc ctaaagcaag gaaccccacc gtgacgtacg ggaaaaacca
2821 agtcatcatg ctactgtatc ctgaccaccc aacactcctg tcctaccgga atatgggaga
2881 agaaccaaac tatcaagaag agtgggtgat gcataagaag gaagtcgtgc taaccgtgcc
2941 gactgaaggg ctcgaggtca cgtggggcaa caacgagccg tataagtatt ggccgcagtt
3001 atctacaaac ggtacagccc atggccaccc gcatgagata attctgtatt attatgagct
3061 gtaccccact atgactgtag tagttgtgtc agtggccacg ttcatactcc tgtcgatggt
3121 gggtatggca gcggggatgt gcatgtgtgc acgacgcaga tgcatcacac cgtatgaact
3181 gacaccagga gctaccgtcc ctttcctgct tagcctaata tgctgcatca gaacagctaa
3241 agcggccaca taccaagagg ctgcgatata cctgtggaac gagcagcaac ctttgttttg
3301 gctacaagcc cttattccgc tggcagccct gattgttcta tgcaactgtc tgagactctt
3361 accatgctgc tgtaaaacgt tggctttttt agccgtaatg agcgtcggtg cccacactgt
3421 gagcgcgtac gaacacgtaa cagtgatccc gaacacggtg ggagtaccgt ataagactct
3481 agtcaataga cctggctaca gccccatggt attggagatg gaactactgt cagtcacttt
3541 ggagccaaca ctatcgcttg attacatcac gtgcgagtac aaaaccgtca tcccgtctcc
3601 gtacgtgaag tgctgcggta cagcagagtg caaggacaaa aacctacctg actacagctg
3661 taaggtcttc accggcgtct acccatttat gtggggcggc gcctactgct tctgcgacgc
3721 tgaaaacacg cagttgagcg aagcacacgt ggagaagtcc gaatcatgca aaacagaatt
3781 tgcatcagca tacagggctc ataccgcatc tgcatcagct aagctccgcg tcctttacca
3841 aggaaataac atcactgtaa ctgcctatgc aaacggcgac catgccgtca cagttaagga
3901 cgccaaattc attgtggggc caatgtcttc agcctggaca cctttcgaca acaaaattgt
3961 ggtgtacaaa ggtgacgtct ataacatgga ctacccgccc tttggcgcag gaagaccagg
4021 acaatttggc gatatccaaa gtcgcacacc tgagagtaaa gacgtctatg ctaatacaca
4081 actggtactg cagagaccgg ctgtgggtac ggtacacgtg ccatactctc aggcaccatc
4141 tggctttaag tattggctaa aagaacgcgg ggcgtcgctg cagcacacag caccatttgg
4201 ctgccaaata gcaacaaacc cggtaagagc ggtgaactgc gccgtaggga acatgcccat
4261 ctccatcgac ataccggaag cggccttcac tagggtcgtc gacgcgccct ctttaacgga
4321 catgtcgtgc gaggtaccag cctgcaccca ttcctcagac tttgggggcg tcgccattat
4381 taaatatgca gccagcaaga aaggcaagtg tgcggtgcat tcgatgacta acgccgtcac
4441 tattcgggaa gctgagatag aagttgaagg gaattctcag ctgcaaatct ctttctcgac
4501 ggccttagcc agcgccgaat tccgcgtaca agtctgttct acacaagtac actgtgcagc
4561 cgagtgccac cccccgaagg accacatagt caactacccg gcgtcacata ccaccctcgg
4621 ggtccaggac atctccgcta cggcgatgtc atgggtgcag aagatcacgg gaggtgtggg
4681 actggttgtt gctgttgccg cactgattct aatcgtggtg ctatgcgtgt cgttcagcag
4741 gcactaattt ttatcctgca ggacttcgaa accgttattt atagcagata tagggatagg
4801 agtaggaatg ccacaaatga aaaaaatact taaaatgtaa tcttaatcga gtacaccaca
4861 cgacaatgaa caaacctaag acagattatg ctggttatgc ttgctgcgta atatgcggtc
4921 taattgtcgg aattattttt acagcgacac tattaaaagt tgtagaacgt aaattagttc
4981 atacaccatc aatagataaa acgataaaag atgcatatat tagagaagat tgtcctactg
5041 actggataag ctataataat aaatgtatcc atttatctac tgatcgaaaa acctgggagg
5101 aaggacgtaa tacatgcaaa gctctaaatc caaattcgga tctaattaag atagagactc
5161 caaacgagtt aagtttttta agaagcctta gacgaggcta ttgggtagga gaatccgaaa
5221 tattaaacca gacaacccca tataatttta tagctaaaaa tgccacgaag aatggaaata

```
     5281 tatttgtagc acaacgaata ctcccaaact gcattcgtgt tacactatat aacaattaca
     5341 ctacattttt atcataccac tacttcggtt agatgtttta g
//
```

ZIKV-PrME immunogen genes located between B6R and B9R ORFs

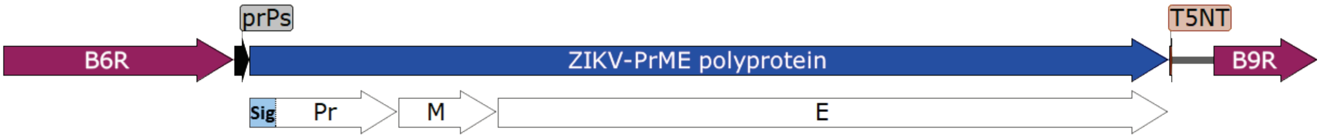


```
LOCUS       Exported                2968 bp ds-DNA     linear   UNA 27-FEB-2018
DEFINITION  ZIKV-PrME expression cassette within SCV1002.
ACCESSION   .
VERSION     .
KEYWORDS    .
SOURCE      SCV1002
  ORGANISM  SCV1002
REFERENCE   1  (bases 1 to 2968)
  AUTHORS   Paul Howley
  TITLE     Direct Submission
  JOURNAL   Exported Feb 27, 2018 from SnapGene 4.1.5
            http://www.snapgene.com
COMMENT     ZIKV-PrME expression cassette was inserted by homologous
            recombination to replace both B7R and B8R ORFs
FEATURES             Location/Qualifiers
     source          1..2968
                     /organism="SCV1002"
                     /strain="Copenhagen"
                     /mol_type="genomic DNA"
     CDS             1..522
                     /codon_start=1
                     /product="putative protein"
                     /label=B6R
                     /note="VACV-COP-B6R ORF"
                     /translation="MSSSVDVDIYDAVRAFLLRHYYNKRFIVYGRSNAILHNIYRLFTR
                     CAVIPFDDIVRTMPNESRVKQWVMDTLNGIMMNERDVSVSVGTGILFMEMFFDYNKNSI
                     NNQLMYDIINSVSIILANERYRSAFNDDGIYIRRNMINKLYGYASLTTIGTIAGGVCYY
                     LLMHLVSLYK"
     promoter        523..557
                     /label=prPs
                     /note="Vaccinia virus early/late promoter"
     CDS             558..2633
                     /codon_start=1
                     /product="PrME polyprotein"
                     /label=ZIKV-PrME polyprotein
                     /note="ZIKV-PrME polyprotein coding sequence from the
                     Brazilian ZikaSPH2015 isolate (Genbank: KU321639)"
                     /translation="MGADTSVGIVGLLLTTAMAAEVTRRGSAYYMYLDRNDAGEAISFP
                     TTLGMNKCYIQIMDLGHMCDATMSYECPMLDEGVEPDDVDCWCNTTSTWVVYGTCHHKK
                     GEARRSRRAVTLPSHSTRKLQTRSQTWLESREYTKHLIRVENWIFRNPGFALAAAAIAW
                     LLGSSTSQKVIYLVMILLIAPAYSIRCIGVSNRDFVEGMSGGTWVDIVLEHGGCVTVMA
                     QDKPTVDIELVTTTVSNMAEVRSYCYEASISDMASDSRCPTQGEAYLDKQSDTQYVCKR
                     TLVDRGWGNGCGLFGKGSLVTCAKFACSKKMTGKSIQPENLEYRIMLSVHGSQHSGMIV
                     NDTGHETDENRAKVEITPNSPRAEATLGGFGSLGLDCEPRTGLDFSDLYYLTMNNKHWL
                     VHKEWFHDIPLPWHAGADTGTPHWNNKEALVEFKDAHAKRQTVVVLGSQEGAVHTALAG
                     ALEAEMDGAKGRLSSGHLKCRLKMDKLRLKGVSYSLCTAAFTFTKIPAETLHGTVTVEV
                     QYAGTDGPCKVPAQMAVDMQTLTPVGRLITANPVITESTENSKMMLELDPPFGDSYIVI
                     GVGEKKITHHWHRSGSTIGKAFEATVRGAKRMAVLGDTAWDFGSVGGALNSLGKGIHQI
```

```
                     FGAAFKSLFGGMSWFSQILIGTLLMWLGLNTKNGSISLMCLALGGVLIFLSTAVSA"
     mat_peptide     558..893
                     /product="propeptide with N-terminal signal sequence "
                     /label=Pr
                     /note="ZIKV propeptide with N-terminal signal sequence
                     coding sequence"
     mat_peptide     894..1118
                     /product="membane protein"
                     /label=M
                     /note="ZIKV mature Membrane protein coding sequence"
     mat_peptide     1119..2633
                     /product="envelope glycoprotein"
                     /label=E
                     /note="ZIKV envelope protein coding sequence"
     misc_feature    2634..2640
                     /label=T5NT
                     /note="Poxvirus early transcriptional stop sequence"
     CDS             2735..2968
                     /codon_start=1
                     /product="6 kDa intracellular protein; non-essential for
                     virus replication and virulence"
                     /label=B9R
                     /note="VACV-COP-B9R ORF"
                     /translation="MRSLIIVLLFPSIIYSMSIRRCEKTEEETWGLKIGLCIIAKDFYP
                     ERTDCSVHLPTASEGLITEGNGFRDIRNTDKL"
ORIGIN
        1 atgtcttctt cagtggatgt tgatatctac gatgccgtta gagcattttt actcaggcac
       61 tattataaca agagatttat tgtgtatgga agaagtaacg ccatattaca taatatatac
      121 aggctattta caagatgcgc cgttataccg ttcgatgata tagtacgtac tatgccaaat
      181 gaatcacgtg ttaaacaatg ggtgatggat acacttaatg gtataatgat gaatgaacgc
      241 gatgtttctg taagcgttgg caccggaata ctattcatgg aaatgttttt cgattacaat
      301 aaaaatagta tcaacaatca actaatgtat gatataatta atagcgtatc tataattcta
      361 gctaatgaga gatatagaag cgcttttaac gacgatggta tatacatccg tagaaatatg
      421 attaacaagt tgtacggata cgcatctcta actactattg gcacgatcgc tggaggtgtt
      481 tgttattatc tgttgatgca tctagttagt ttgtataaat aaattgaaat tttatttttt
      541 ttttttggaa tataaatatg ggcgcagata ctagtgtcgg aattgttggc ctcctgctga
      601 ccacagctat ggcagcggag gtcactagac gtgggagtgc atactatatg tacttggaca
      661 gaaacgatgc tggggaggcc atatcttttc caaccacatt ggggatgaat aagtgttata
      721 tacagatcat ggatcttgga cacatgtgtg atgccaccat gagctatgaa tgccctatgc
      781 tggatgaggg ggtggaacca gatgacgtcg attgttggtg caacacgacg tcaacttggg
      841 ttgtgtacgg aacctgccat cacaaaaaag gtgaagcacg gagatctaga agagctgtga
      901 cgctcccctc ccattccact aggaagctgc aaacgcggtc gcaaacctgg ttggaatcaa
      961 gagaatacac aaagcacttg attagagtcg aaaattggat attcaggaac cctggcttcg
     1021 cgttagcagc agctgccatc gcttggcttt tgggaagctc aacgagccaa aaagtcatat
     1081 acttggtcat gatactgctg attgccccgg catacagcat caggtgcata ggagtcagca
     1141 atagggactt tgtggaaggt atgtcaggtg ggacttgggt tgatattgtc ttggaacatg
     1201 gaggttgtgt caccgtaatg gcacaggaca aaccgactgt cgacatagag ctggttacaa
     1261 caacagtcag caacatggcg gaggtaagat cctactgcta tgaggcatca atatcagaca
     1321 tggcttcgga cagccgctgc ccaacacaag gtgaagccta ccttgacaag caatcagaca
     1381 ctcaatatgt ctgcaaaaga acgttagtgg acagaggctg gggaaatgga tgtggacttt
     1441 ttggcaaagg gagtctggtg acatgcgcta agtttgcatg ctccaagaaa atgaccggga
     1501 agagcatcca gccagagaat ctggagtacc ggataatgct gtcagttcat ggctcccagc
     1561 acagtgggat gatcgttaat gacacaggac atgaaactga tgagaataga gcgaaggttg
     1621 agataacgcc caattcacca agagccgaag ccaccctggg gggttttgga agcctaggac
     1681 ttgattgtga accgaggaca ggccttgact tttcagattt gtattacttg actatgaata
     1741 acaagcactg gttggttcac aaggagtggt tccacgacat tccattacct tggcacgctg
     1801 gggcagacac cggaactcca cactggaaca acaaagaagc actggtagag ttcaaggacg
     1861 cacatgccaa aaggcaaact gtcgtggttc tagggagtca agaaggagca gttcacacgg
     1921 cccttgctgg agctctggag gctgagatgg atggtgcaaa gggaaggctg tcctctggcc
     1981 acttgaaatg tcgcctgaaa atggataaac ttagattgaa gggcgtgtca tactccttgt
     2041 gtaccgcagc gttcacattc accaagatcc cggctgaaac actgcacggg acagtcacag
     2101 tggaggtaca gtacgcaggg acagatggac cttgcaaggt tccagctcag atggcggtgg
     2161 acatgcaaac tctgacccca gttgggaggt tgataaccgc taaccccgta atcactgaaa
     2221 gcactgagaa ctctaagatg atgctggaac ttgatccacc atttggggac tcttacattg
     2281 tcataggagt cggggagaag aagatcaccc accactggca caggagtggc agcaccattg
     2341 gaaaagcatt tgaagccact gtgagaggtg ccaagagaat ggcagtcttg ggagacacag
     2401 cctgggactt tggatcagtt ggaggcgctc tcaactcatt gggcaagggc atccatcaaa
     2461 tttttggagc agctttcaaa tcattgtttg gaggaatgtc ctggttctca caaattctca
```

```
     2521 ttggaacgtt gctgatgtgg ttgggtctga acacaaagaa tggatctatt tcccttatgt
     2581 gcttggcctt agggggagtg ttgatcttct tatccacagc cgtctctgct taattttat
     2641 ttaattaact aatttttatt aatgatacaa aaacgaaata aaactgcata ttatacactg
     2701 gttaacgccc ttataggctc taaccatttt caagatgagg tccctgatta tagtccttct
     2761 gttccccctct atcatctact ccatgtctat tagacgatgt gagaagactg aagaggaaac
     2821 atggggattg aaaatagggt tgtgtataat tgccaaagat ttctatcccg aaagaactga
     2881 ttgcagtgtt catctcccaa ctgcaagtga aggattgata actgaaggca atggattcag
     2941 ggatatacga aacaccgata aattataa
//
```

D13L deletion site

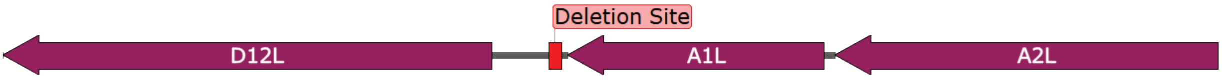


```
LOCUS       Exported                2146 bp ds-DNA     linear   UNA 27-FEB-2018
DEFINITION  D13L deletion site of SCV1002.
ACCESSION   .
VERSION     .
KEYWORDS    .
SOURCE      SCV1002
  ORGANISM  SCV1002
REFERENCE   1  (bases 1 to 2146)
  AUTHORS   Paul Howley
  TITLE     Direct Submission
  JOURNAL   Exported Feb 27, 2018 from SnapGene 4.1.5
            http://www.snapgene.com
COMMENT     The D13L ORF was deleted by homologous recombination and rescue in a
            cell line expressing in trans the D13 protein
FEATURES             Location/Qualifiers
     source          1..2146
                     /organism="SCV1002"
                     /strain="Copenhagen"
                     /mol_type="genomic DNA"
     CDS             complement(1..864)
                     /codon_start=1
                     /product="mRNA capping enzyme small subunit;
                     methyltransferase"
                     /label=D12L
                     /note="VACV-COP-D12L"
                     /translation="MDEIVKNIREGTHVLLPFYETLPELNLSLGKSPLPSLEYGANYFL
                     QISRVNDLNRMPTDMLKLFTHDIMLPESDLDKVYEILKINSVKYYGRSTKADAVVADLS
                     ARNKLFKRERDAIKSNNHLTENNLYISDYKMLTFDVFRPLFDFVNEKYCIIKLPTLFGR
                     GVIDTMRIYCSLFKNVRLLKCVSDSWLKDSAIMVASDVCKKNLDLFMSHVKSVTKSSSW
                     KDVNSVQFSILNDPVDTEFINKFLEFSNRVYEALYYVHSLLYSSMTSDSKSIENKHQRR
                     LVKLLL"
     misc_feature    965..988
                     /label=Deletion Site
                     /note="Remnants of the D13L deleted ORF"
     CDS             complement(999..1451)
                     /codon_start=1
                     /product=" late gene transcription factor VLTF-2"
                     /label=A1L
                     /note="VACV-COP-A1L"
                     /translation="MAKRVSLPDVVISAPKAVFKPAKEEALACILPKYYKSMADVSIKT
                     NSVIDKCWFCNQDLVFRPISIETYKGGEVGYFCSKICRDSLASMVKSHVALREEPKISL
                     LPLVFYEDKEKVINTINLLRDKDGVYGSCYFKENSQIIDISLRSLL"
```

```
     CDS             complement(1472..2146)
                     /codon_start=1
                     /product="late gene transcription factor VLTF-3"
                     /label=A2L
                     /note="VACV-COP-A2L ORF"
                     /translation="MNLRLCSGCRHNGIVSEQGYEYCIFCESVFQKCTKVQKKSNFHVS
                     NKLIHLRNVLRRLLSHQCSGEIISELLDIMEKNQISTDDVDANFVSSFLKANERINKKD
                     YKLVFEIINQVKDEKLNLSTEKINEVVEIFKHLVFFCQENTPSKTINYSFFLDKIFDIT
                     SVTKNLKPQTVKNYTKNNSNQLVWENFLAHMRSKKRVTMVEDYGHEYVFVDERFSTCSL
                     EV"
ORIGIN
        1 tcacagcagt agtttaacta gtcttctctg atgtttgttt tcgatacttt tcgaatcaga
       61 agtcatacta gaataaagca gcgagtgaac gtaatagaga gcttcgtata ctctattcga
      121 aaactctaag aacttattaa tgaattccgt atccactgga tcgtttaaaa tactaaattg
      181 aacactgttc acatccttcc aagaagaaga cttagtgacg gacttaacat gagacataaa
      241 taaatccaaa tttttttac aaacatcact agccaccata atggcgctat ctttcaacca
      301 gctatcgctt acgcatttta gcagtctaac atttttaaag agactacaat atattctcat
      361 agtatcgatt acacctctac cgaataaagt tggaagttta ataatacaat atttttcgtt
      421 tacaaaatca aataatggtc gaaacacgtc gaaggttaac atcttataat cgctaatgta
      481 tagattgttt tcagtgagat gattattaga tttaatagca tctcgttcac gtttgaacag
      541 tttattgcgt gcgctgaggt cggcaactac ggcgtccgct ttagtactcc tcccataata
      601 ctttacgcta ttaatcttta aaatttcata gactttatct agatcgcttt ctggtaacat
      661 gatatcatgt gtaaaaagtt ttaacatgtc ggtcggcatt ctatttagat cattaactct
      721 agaaatctga agaaagtaat tagctccgta ttccagacta ggtaatgggc ttttacctag
      781 agacagatta agttctggca atgtttcata aaatggaaga aggacatgcg ttccctcccg
      841 gatattttt acaatttcat ccatttacaa ctctatagtt tgttttcatt attattagtt
      901 attatctccc ataatcttgg taatacttac cccttgatcg taagatacct tatacaggtc
      961 attacctgca ggttaattaa atttaaattg gaaaaagctt acaataaact ccgtagagaa
     1021 atatctataa tttgtgagtt ttccttaaag taacagcttc cgtaaacgcc gtctttatct
     1081 cttagtaagt ttattgtatt tataaccttt tccttatctt catagaatac taaaggcaac
     1141 aaagaaattt ttggttcttc tctaagagct acgtgagact taaccataga agccaacgaa
     1201 tccctacata ttttagaaca gaaataccct acttcaccac ccttgtatgt ctcaatacta
     1261 ataggtctaa aaaccaaatc ttgattacaa aaccaacact tatcaattac actatttgtc
     1321 ttaatagaca catctgccat agatttataa tactttggta gtatacaagc gagtgcttct
     1381 tctttagcgg gcttaaagac tgctttaggt gctgaaataa ccacatctgg aaggcttact
     1441 cgcttagcca tttaattacg gaactatttt tttatacttc taatgagcaa gtagaaaacc
     1501 tctcatctac aaaaacgtac tcgtgtccat aatcctctac catagtaaca cgttttttag
     1561 atctcatatg tgctaaaaag ttttcccata ctaattggtt actattattt ttcgtataat
     1621 ttttaacagt ttgaggtttt agatttttag ttacagaagt gatatcgaat attttatcca
     1681 aaaagaatga gtaattaatt gtcttagaag gagtgttttc ttggcaaaag aataccaagt
     1741 gcttaaatat ttctactact tcattaatct ttctgtact cagattcagt ttctcatctt
     1801 ttacttgatt gattatttca aagactaact tataatcctt tttatttatt ctctcgttag
     1861 ccttaagaaa actagataca aaatttgcat ctacatcatc cgtggatatt tgattttttt
     1921 ccatgatatc caagagttcc gagataattt ctccagaaca ttgatgagac aataatctcc
     1981 gcaatacatt tctcaaatga ataagtttat tagacacgtg gaagtttgac tttttttgta
     2041 cctttgtaca tttttgaaat accgactcgc aaaaaataca atattcatat ccttgttcag
     2101 atactatacc gttgtgtcta caaccgctac ataatcgtag attcat
```

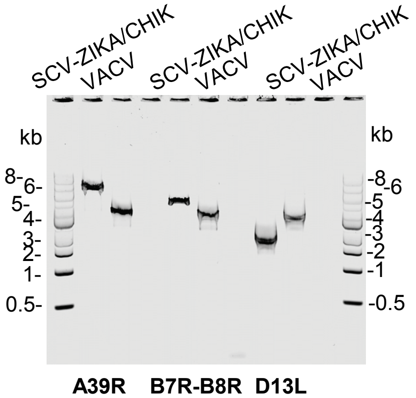

Corresponds to Figure 1c

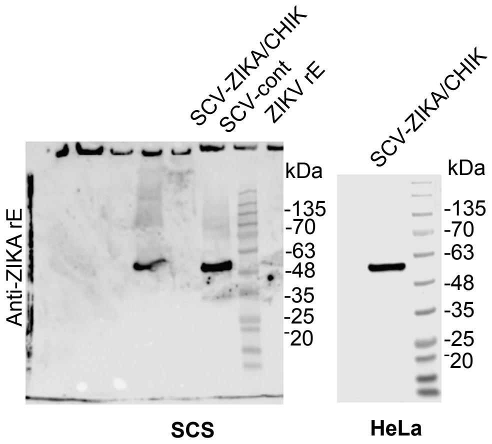

Corresponds to Figure 1d

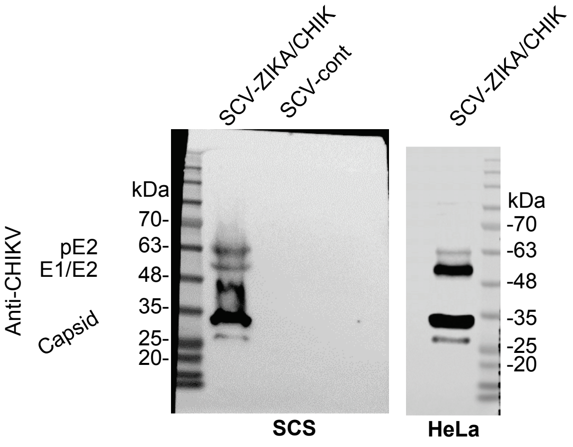

Corresponds to Figure 1e

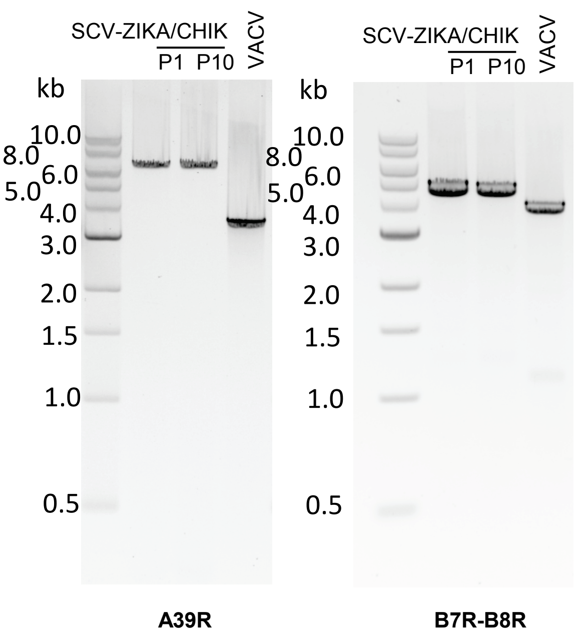

Corresponds to Figure 1f

Supplementary Fig. 4. Uncropped Western blots and PCR gel images for Fig. 1.
